# Views of research ethics committee members on end-of-participation communications for trial participants who stop taking part: a cross-sectional survey study

**DOI:** 10.1186/s13063-024-08465-3

**Published:** 2024-09-30

**Authors:** William J. Cragg, Liam Bishop, Rachael Gilberts, Michael Gregg, Mary Mancini, Clara Martins de Barros

**Affiliations:** 1 Clinical Trials Research Unit, Leeds Institute of Clinical Trials Research, University of Leeds, Leeds, UK; 2 Public Contributor, Leeds, UK

**Keywords:** Participant withdrawal, Participation changes, Participant communication, Ethics review processes

## Abstract

**Background:**

Giving information to trial participants who stop taking part could support them through what can be a difficult process. We previously developed guidance around the ethical acceptability of such information provision, and about how trialists can develop suitable communication materials. There is limited evidence about what research ethics committees think of this issue, and limited guidance about what level of oversight they should have over the proposed communications, or post-consent participant communications generally. We conducted a survey of UK ethics committee members to address these points.

**Methods:**

The survey was co-developed by public contributors and trialists who had previously worked together on the communications guidance. We asked respondents if they agreed with the general idea of informing participants who stop taking part, if they had ever been requested to review similar communications, and what level of ethics committee review they might recommend. The survey was primarily conducted online. It was reviewed by three ethics committee members before finalisation and shared directly with all UK ethics committee members. We analysed quantitative questions descriptively and used inductive analysis for open questions to identify common themes.

**Results:**

Ninety-one ethics committee members participated (nearly 10% of all UK members). The sample was similar to reported data about all members in terms of several personal characteristics. Most respondents (83%) agreed with our project’s rationale. Only 23% of respondents reported having been asked to review an end-of-participation information sheet before. Respondents gave various answers about the level of ethics committee review required, but most supported a relatively proportionate review process. Common concerns were about the risk of coercion or making participants feel pressured.

**Conclusions:**

Our survey suggests that ethics committee members generally support providing information to trial participants who stop taking part, if risks to participants are mitigated. We believe our guidance already addresses the main concerns raised. Our respondents’ lack of prior experience with end-of-participation information sheets suggests that participants are not getting information they want or need when they stop participating. Our results help clarify how ethics committee should oversee post-consent participant communications, but further guidance from research regulators could be helpful.

**Supplementary Information:**

The online version contains supplementary material available at 10.1186/s13063-024-08465-3.

## Background

Participants in clinical trials have the right to stop taking part at any time. There is evidence that the process of stopping can sometimes be a stressful or difficult experience for them [1, 2]. Providing participants with information they might want or need at the time of stopping could help reassure them about their decision to stop and clarify about what will happen next for them and their care.

Anecdotally, we have heard research staff express uncertainty about whether it is acceptable to communicate with participants once they have ‘withdrawn consent’, perhaps motivated by a desire to avoid any perceptions of coercion or trying to change participants’ minds. The PeRSEVERE project (PRincipleS for handling end-of-participation EVEnts in clinical trials REsearch [3]) aimed to address this and other uncertainties by establishing guiding principles for how participation changes should be managed in clinical trials and other research. One of the principles (coded ‘O7’) established the idea that it is acceptable and indeed ethical to provide participants with information they might want or need after they stop taking part. From our experience and through engaging with various research staff through PeRSEVERE and related work, it seems likely that providing end-of-participation information is quite rare.

To provide more practical guidance about how this sort of information provision can be achieved, a group of public contributors and trialists at the University of Leeds Clinical Trials Research Unit (CTRU) produced researcher guidance with example wording [4]. The details of this work have been published separately [5].

Given the sensitivity around participants’ freedom to withdraw their consent, many may agree that an end-of-participation communication needs suitable oversight from an independent research ethics committee (REC), perhaps ideally as part of initial project submission. Understanding the views of REC members is therefore an important part of assessing the feasibility of this sort of communication. If REC members find end-of-participation communications ethically problematic then it may be harder for trialists to implement them in their trials.

There is limited evidence available about what REC members think about participant communication after initial consent in general and how much oversight they would like to have. The REC Standard Operating Procedure [6] does not give a definitive statement about expectations for REC review of participant-facing communications, i.e. whether all communications should be REC approved before use, and if so, what sort of details need to be reviewed.

UK Health Research Authority (HRA) has released guidance about informing participants at the end of a study [7]. This covers expectations around ethical review of information materials. The guidance takes a proportionate approach, saying that end of study information sheets do not need additional REC review if they ‘[build] on the information provided in the original [patient information sheet] and [are] in line with the arrangements agreed by the REC as part of [original study approval]’. The HRA also collaborated with Parkinson’s UK on a toolkit for ‘staying connected with your participants’ [8]. This emphasises obtaining approval for participant-facing materials as part of initial trial submissions, but this does not therefore cover expectations for materials tailored to each individual’s circumstances (as end of participation materials may ideally be).

Neither of these resources say anything about informing participants who stop taking part early, at the time they stop (as opposed to informing them at the global trial end, for example). Participants who stop taking part early arguably have distinct information needs. Informing these participants poses specific ethical issues (given they have ‘withdrawn their consent’ at that stage) that are different to those around general end of study communications.

It might be that RECs are more concerned with communications that have implications for initial or ongoing consent, and less so on simple updates for participants, but this position is not explicit in guidance from the HRA. Our proposed end-of-participation information sheet incorporates elements of both these areas: it serves to update participants but is also relevant to participants’ consent (or rather their decision to withdraw or change their consent). Some level of REC review therefore seems important. It might also give researchers reassurance about this relatively unexplored form of participant communication.

We conducted a short, cross-sectional survey of UK REC members to find out their views on the topic of end-of-participation communications and what RECs’ role should be.

## Methods

### Survey objectives

The cross-sectional survey aimed:

1. To find out how much UK REC members support the general idea of researchers communicating with participants who have stopped taking part in a trial or other study.
2. To establish if UK REC members tend to have reservations about this sort of communication and whether we needed to amend our existing guidance to address such reservations.

We have used a Checklist for Reporting of Survey Studies (CROSS) [9] to ensure complete reporting (see Supplementary Information).

### Survey design

A subgroup of the main group working on the researcher guidance, including trialist and public contributors, devised and agreed the survey questions. A full copy of the questionnaire text is available as Supplementary Information. The main quantitative questions asked REC members:

- The extent to which they agreed with the underlying rationale for our project (with the rationale presented on-screen first).
- Whether they had previously been asked for an opinion on written communications for research participants who stop taking part.
- What level of REC review and oversight might be appropriate for this sort of communication.

We used additional open questions to explore rationale for the quantitative responses and to gather feedback on any concerns among REC members about approving use of our proposed end-of-participation communications. A series of additional questions collected some information about the respondents, namely the region of the UK where their REC is based, their length of time spent as a REC member, their role on the REC and their age, gender and ethnicity.

We conducted the survey primarily online using the Jisc Online Surveys platform [10]. We also provided other routes for those who did not want to use the online option for any reason. The other methods offered included completing a paper copy of the survey, or responding to questions over the phone or via an online meeting, or any other feasible method depending on the respondent’s preferences or needs.

The survey was reviewed by three current or former REC members before it was finalised, including one lay member.

### Eligibility and recruitment

Individuals were eligible if they were currently a decision-making member of an HRA-managed REC in the UK. We excluded former REC members as we were interested in views of members who might review current or future REC submissions. We excluded people with administrative roles as we wanted to know the views of those involved in decision-making. We made clear in the survey introduction that we wanted people to contribute as individuals, rather than on behalf of their REC.

The survey invitation message and link were shared directly with REC members by the HRA and in the regular REC member newsletter. One reminder message was also sent via this route. We also shared details via X (formerly Twitter) and via the UK Trial Managers’ Network [11], which we considered somewhat likely to contain REC members.

In line with HRA guidance on proportionate consent [12], we presumed that respondents gave consent to participate in the survey when they chose to complete it. This was made clear in the survey introduction text, alongside adequate information about the survey, implications of taking part, and their right to stop taking part before completing the survey without negative consequences.

The survey did not ask for any identifiable personal or confidential data. Respondents’ identities would need to be revealed to the project lead if they wanted to complete the survey outside of the online platform, but nonetheless no one’s identity was recorded in the survey. We set up a mailing list for individuals to receive the survey results directly, but this was not linked to the survey responses.

Due to the exploratory nature of this work, we did not calculate a sample size. Instead, we aimed to collect as many responses as possible, with ideally a good balance of individuals from different groups (based on the questions about respondents’ characteristics). We closed the survey when the planned dissemination was complete and it was clear that the chance of further responses was unlikely.

Full details of the survey plan and invitation message are available as Supplementary Information.

### Data handling and analysis

The project lead (WJC) managed and analysed the survey data using Microsoft Excel. He performed a check for possible duplicate responses and for any inconsistent or illogical responses (e.g. free text comments conflicting with category responses). Any findings from these checks would be discussed with the rest of the team and a suitable action agreed.

We included all respondents in the analysis if they had confirmed they were eligible and had saved at least one response into the survey. We summarised category questions descriptively. We included missing data in the descriptive statistics. We summarised the initial question about agreement with our project’s rationale by converting responses to numbers 1–5 (with ‘strongly disagree’ being 1 and ‘strongly agree’ being 5) in order to calculate a median.

We did not plan to impute any data, except potentially in the situation where a category question was left blank but the related free text response unambiguously indicated an answer (e.g. ‘I completely agree with this’).

Responses to free text questions were summarised inductively, working without a pre-existing framework to categorise comments initially at a granular level (i.e. based on its specific contents) then combining these categories into broader themes. We chose this approach as we had neither a prior framework to work with, nor any strong rationale to make prior assumptions about the sorts of comments we would receive. Three authors (WJC, LB, RG) jointly reviewed a random 10% sample of the comments to check that the coding and resulting themes were representative of the underlying comments received.

We carried out exploratory analysis of the quantitative and qualitative responses to look for potential differences in answers given by different subgroups. We also planned to review responses by response method, if a substantial number of people responded outside of the default online route. These exploratory analyses only involved reviewing descriptive statistics for each subgroup to see if there might be a potential difference worthy of further, future research. There was no planned statistical testing of any identified differences.

When the results were complete, we shared a summary directly with respondents who had signed up to the mailing list and with HRA for inclusion in the REC member newsletter.

### Patient and public involvement

This survey arose from a patient and public involvement activity. Members of the broader group working on that project were invited to help with this work. Four public contributors (three of whom are co-authors on this paper) accepted the offer and helped to devise the survey questions, agree the survey dissemination details, and review and interpret the results. A patient member of a REC also reviewed the draft survey.

## Results

### Respondent characteristics and views on proposed participant communication

Ninety-one REC members completed the survey between 15/12/2022 and 07/03/2023. Based on information from HRA, this equates to 8% of all current REC members (total 1130), but this included responses from around 17% of REC chairs. All 91 respondents completed all three of the main quantitative questions.

See Table 1 for details of respondents’ characteristics. Respondents were from across all regions of the UK except for Northern Ireland. Many of the respondents were female, over 45 years old and described themselves as having white ethnicity. This broadly aligns with HRA’s published figures on their membership’s characteristics [13].

**Table 1:** Survey respondents’ characteristics

	***n***	**%**
**Which region is your REC in?**		
England—East Midlands	7	8%
England—East of England	7	8%
England—London	24	26%
England—North East	5	5%
England—North West	4	4%
England—South Central	7	8%
England—South West	7	8%
England—West Midlands	6	7%
England—Yorkshire and the Humber	11	12%
Northern Ireland	0	0%
Scotland	7	8%
Wales	5	5%
Not sure/none of the above	1	1%
**How long have you been a REC member (on any REC)?**		
Less than 1 year	13	14%
1–5 years	41	45%
6 or more years	36	40%
Not sure/other	0	0%
[Missing]	1	1%
**What is your role on the ethics committee?**		
Chair	15	16%
Vice chair	11	12%
Lay member	18	20%
Lay plus member	13	14%
Expert member	33	36%
Not sure/other	0	0%
[Missing]	1	1%
**How old are you?**		
Younger than 30 years old	1	1%
30–45 years old	17	19%
46–65 years old	38	42%
66 + years old	33	36%
Prefer not to say	2	2%
**How would you describe your gender?**		
Female	51	56%
Male	39	43%
Non-binary	0	0%
None of the above categories	0	0%
Prefer not to say	1	1%
**How would you describe your ethnicity?**		
Asian	2	2%
Black	1	1%
Mixed or multiple ethnicities	1	1%
White	85	93%
None of the above categories	0	0%
Prefer not to say	2	2%

**Fig. 1:**
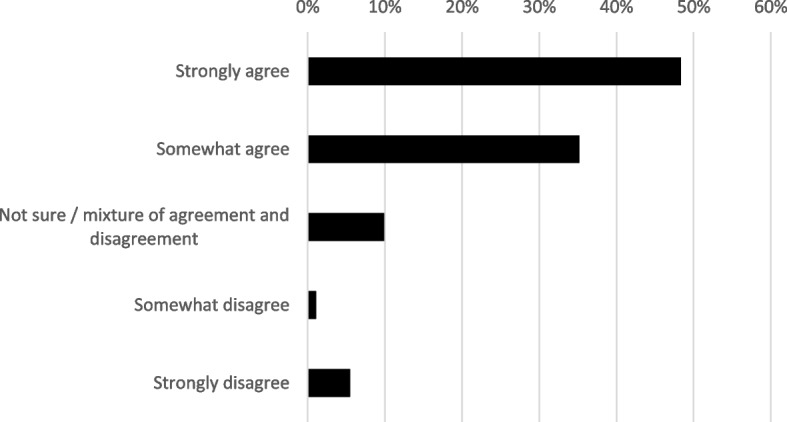
Extent of survey respondents’ (*n* = 91) agreement with rationale behind our communications guidance^a^. ^a^The rationale explained why we consider it important to provide information to participants around the time they stop taking part, and the sorts of information we suggest should be provided. See Supplementary Information for full text that was shown to respondents

Most respondents (83%) agreed or strongly agreed with the rationale for our project. The median response was 4, i.e. ‘agree’. Only 6% of respondents somewhat or strongly disagreed (Fig. 1). The most common reasons for supporting our rationale were support for particular types of information we had mentioned (e.g. confirmation of how participation has changed or information about what happens next), general support for the idea of keeping participants informed, and support for the idea of preventing participants feeling abandoned when they stop taking part in a trial. The most common cautious or negative comments were about the potential for the end-of-participation communication to be coercive, or for participants to feel harassed or that their right to withdraw consent was being compromised. There were also comments about the need for participants to be able to choose to have no further contact when they stop, and about the challenges to adjust information to study type, participants’ circumstances or the timing of their stopping.

**Fig. 2:**
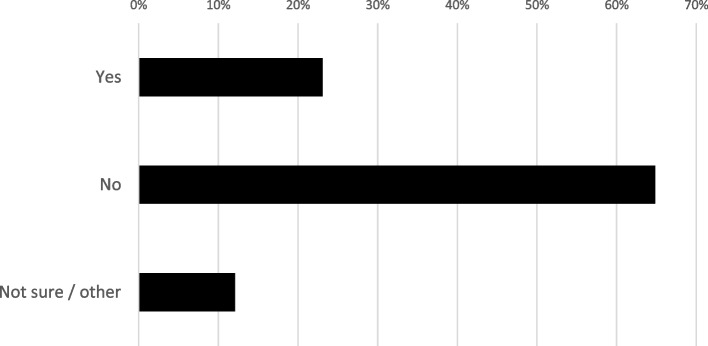
Whether survey respondents (*n* = 91) had ever been asked to review end-of-participation communications for early-stopping participants

Only 23% of respondents said they had been asked to review this sort of communication before (Fig. 2), confirming that this is not commonly done at present.

### Potential concerns about the approach

In our open question about whether respondents would have concerns about the approach, 48 respondents (53%) raised at least one potential concern. The most common response was about the possibility of participants feeling pressured by receiving the communication—including suggestions that any contact at all might be unwelcome for some. Others were concerned that participants might be encouraged to change their mind about stopping participation, or might be asked to explain their decision to stop. Other concerns were about the content and quality of the communications, including aspects such as accessibility, tone, or wording choice.

### Views on the REC review process

**Fig. 3:**
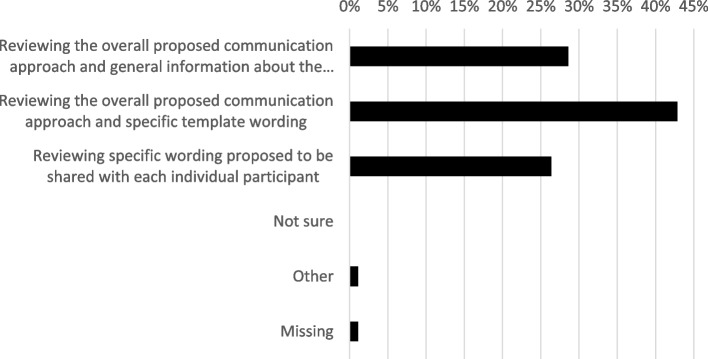
Level of ethics committee review that respondents (*n* = 91) recommend for end-of-participation communications for early-stopping participants

Regarding how RECs should review and oversee our proposed communication (Fig. 3), the largest group of respondents (43%) supported REC reviews of the overall proposed approach and template wording, but not review of the wording to be shared with each individual participant. Smaller groups were in favour of RECs reviewing only the overall approach with the topics that would be included (29%) or reviewing specific wording intended for each participant (26%). The free text comments suggested that some of the respondents may not have fully understood the distinction between the template wording and specific wording options, and mainly wanted to express a view that RECs should review the wording that would be used (whether personalised or not) rather than just topics that would be included.

In explaining their responses, people in favour of a lighter-touch review suggested that trialists need flexibility to adjust their approach to different participants or situations. Others said that RECs should trust trialists to communicate with participants, and that a check of what sorts of content would be included would be enough.

Those in favour of the template wording option mentioned wanting to check wording would not be upsetting, or make participants feel guilty or pressured. Others said they wanted to check the accessibility and clarity of documents, and how it aligned with what else participants had been told. Some mentioned that RECs should take a similar approach to review of other participant-facing materials (e.g. initial consent materials) and did not need more oversight than that (even though the end-of-participation communication would be more tailored to each individual than the initial patient information sheet). Some commented that it would not be feasible for RECs to review each individual participant’s communication.

Those who were in favour of the individual-level review mentioned wanting to check that the wording used would not cause upset or make participants feel guilty. Some preferred this option as the ‘general’ review would not be adequate and the template wording review would not give them enough oversight.

### Other comments and exploratory subgroup analyses

We asked if respondents had any other comments on the process for reviewing this proposed communication. Several people mentioned about the timing of review, emphasising that review at the start of a trial would be best, rather than adding it in later. This matches our guidance [14]. Others mentioned that it would be important for reviewing RECs to have a clear idea of the context in which the communication would be given, e.g. who gives it, when it is given and how it fits with other trial communications.

In the exploratory analysis of respondent subgroups, there was some suggestion that REC chairs were more supportive of our guidance and of a lighter-touch REC review, with lay members more cautious. However, overall, there was not a large enough sample to be able to draw any strong conclusions about whether different groups might have different views. Only one person completed the survey outside the online platform, so there was no justification for reviewing results by method of response.

## Discussion

### Implications of the survey findings

The results of our survey suggest that the idea of trialists giving information to trial participants when they stop taking part may be acceptable to many REC members. We will continue to learn more about ethics committee views as we put our guidance into practice, but we hope that, for now, the data presented here will reassure trialists that ethics committees support end-of-participation communications if they are prepared in line with our guidance. We have nonetheless refined our guidance based on the survey results, to more directly address some of the concerns raised.

Survey respondents highlighted some challenges and complexities in doing this sort of communication, and the need for caution to mitigate the risk that participants feel pressured by receiving the communication. We agree with these points, and we had already considered them during development of our guidance. The example wording in our guidance was developed with and by public contributors and was designed to inform but not put pressure on participants. In our suggested process for developing a communication strategy and materials, we emphasise involving public contributors in the process, considering the needs of the participants in the specific study, and assessing each participant’s circumstances before making contact.

Some respondents suggested an end-of-participation information sheet should only be given if participants have consented to receive it. We agree that participants should have choices about how much information they get and should not be surprised by receiving an end-of-participation communication. We also agree with the need to respect individual participants’ wishes for no further contact at all, when they have said (or implied) that this is what they want. However, we suggest that it would cause problems to ask for participants’ consent to receive the information as part of their original consent to take part. If a participant gave that consent but then later said they wanted to stop taking part in the trial—i.e. they withdrew their consent—it might be difficult to know if the original consent to receive the information still stood, or if it had also been withdrawn when they stopped taking part. We therefore advise researchers against asking participants to give consent to receive an end-of-participation communication. See PeRSEVERE project guidance around consent for more about this sort of issue [3].

Although only a small proportion of all REC members completed the survey, we had responses from members of RECs across the UK, a sizeable number of REC chairs and mixture of lay and expert members. Although we cannot say if those who did not take part have substantially different views on this topic, in terms of age, gender and ethnicity, the respondents broadly seem to match the overall population of REC members.

Most respondents had not been asked to review end-of-participation communications before. Given the spread of responses from across different RECs, this seems likely to reflect the infrequency of the approach. It is hypothetically possible that researchers are using communication materials without informing RECs, but this seems unlikely given anecdotal evidence that trialists are cautious and tend to request REC review of participant-facing materials in case of doubt. This result therefore supports the conclusion that it is common for trial participants to receive limited or no information when they stop taking part. This aligns with our experience and with the previous lack of available guidance about end-of-participation communication. We suggest that the experiences of participants who stop taking part would be a good topic for future research, not only to improve those people’s experiences but potentially to improve the quality of clinical trials overall.

A substantial proportion of respondents accepted the idea of REC oversight only of template wording or even review only of the overall approach. This might suggest that these REC members are prepared to trust researchers to contact participants as they see fit. This may also reflect a recognition that RECs cannot feasibly oversee all participant communications. These findings align with recent reflective work from the HRA, where REC members favoured a focus on ‘fundamental issues, rather than being distracted by minor issues (such as phraseology)’ [15]. A separate, Europe-wide study also highlighted REC members’ concerns about the amount of work potentially required for post-approval oversight activities, and about the potential for REC ‘overreach’ and need for trust between RECs and researchers [16].

There is a general acceptance that participants can be supported by having information they might want or need readily available as they move through a study. This has been repeatedly highlighted via the NIHR Participant in Research Experience Survey [17, 18]. Putting this into practice will mean trialists wanting to do more participant communications. If they are under the impression that RECs need to approve all such communications, this might lead to an unmanageable workload for RECs, or trialists erring on the side of not sharing information that participants want or need because the need for REC review (or perceived need) is too big a barrier. We suggest that more guidance on this from HRA or others would be useful.

One further consideration in establishing such guidance may be that trials vary in terms of who is in direct contact with participants. In some cases, participants mostly interact with healthcare professionals and have no direct contact with the trialists at, for example, a clinical trials unit (CTU). Here, written communications between the CTU and participants might be quite rare, so it will be more feasible for REC to review all of them. Where participants are in direct contact with trialists, there may need to be various kinds of ad hoc communication (in both directions) and it does not seem feasible for REC to oversee it all.

### Strengths and limitations

The strengths of our work are that it sheds light onto a previously underexplored topic, i.e. what REC members think about communication with participants who stop taking part and about REC review processes for participant communications in general.

One limitation not mentioned above is that we prioritised keeping the survey brief. There may therefore be more scope to explore this topic in more depth in future. Although we asked some current REC members to test the survey before we opened it to recruitment, there is some chance of some differences in interpretation, and we saw some evidence of this in the question about REC review. We believe this does not affect our overall conclusions. Our survey did not pose a direct question about whether REC approval for end-of-participation communications is needed or not. Although no respondents used the ‘Other’ option in the question about level of REC oversight to say they thought no REC review was needed, we cannot say for sure that no REC members hold this view. Although we asked respondents to reply for themselves, we cannot rule out conformity bias, whereby individuals’ responses were influenced by what they perceived to be the beliefs of their fellow REC members or others.

We might have taken a more intensive approach to double-checking the coding of comments received through the survey. However, we suggest our approach to jointly review a sample of the comments is proportionate and could not substantially affect our conclusions as the comment coding was only intended to explore perceptions alongside the quantitative survey questions.

## Conclusions

Based on this survey of a sample of REC members, there is support for the idea of giving information to trial participants when they stop taking part. Concerns raised by survey respondents align with those we identified and addressed in our guidance. Our survey confirms that this approach is unlikely to be common at present, meaning participants may be missing out on information they want or need. REC members had different views on the level of review and oversight needed, but a large proportion were in favour of lighter touch reviews.

## Supplementary Information

Additional file 1: Supplement 1 Checklist for Reporting Of Survey Studies (CROSS)

Additional file 2: Supplement 2 Full survey text

Additional file 3: Supplement 3 Survey protocol (with invitation message)

## Data Availability

Data collected as part of this work is available for further research on reasonable request. All requests will be reviewed by relevant stakeholders, based on the principles of a controlled access approach. Requests to access data should be made to CTRU-DataAccess@leeds.ac.uk in the first instance.

## Abbreviations

CROSS: Checklist for Reporting of Survey Studies
CTRU: Clinical Trials Research Unit (University of Leeds)
CTU: Clinical trials unit
HRA: Health Research Authority
PeRSEVERE: PRincipleS for handling end-of-participation EVEnts in clinical trials REsearch
REC: Research ethics committee
