# Supplementary material for: Views of research ethics committee members on end-of-participation communications for trial participants who stop taking part: a cross-sectional survey study
Source: Trials. 2024 Sep 30;25:636. doi: 10.1186/s13063-024-08465-3 (PMC11440916; doi:10.1186/s13063-024-08465-3)
Supplement: Supplementary file 2 — Additional file 2: Supplement 2 Full survey text [file 13063_2024_8465_MOESM2_ESM.pdf]

Supplement 2: full survey text

[Survey information sheet version 2.0]

Thank you for your interest in this short survey. Before you decide to complete the survey, it is important for you to understand why we are doing it and what it will involve for you. We do not believe there would be any significant risks from taking part, but please do take time to read the following information carefully and discuss it with others if you wish. Ask us if there is anything that is not clear or if you would like more information. Take time to decide whether or not you wish to take part. You can download a copy of this introductory information **[link added to regular and large text versions].**

It is completely up to you whether or not you take part in this survey. There will be no consequences for you if you do not take part, or start taking part then stop partway through. You do not have to provide a reason if you start the survey but change your mind and stop before the end. You should be aware, though, that once your survey responses have been submitted, it will not be possible to withdraw them because we will not be able to identify you from your responses.

The survey is run by researchers at the University of Leeds, and has nothing to do with the Health Research Authority or your REC.

What is this survey about?

Research participants sometimes stop taking part early, before their participation was originally due to end. This may be for a variety of reasons, including withdrawal of consent. We (a group of patients and University of Leeds researchers) would like to understand what you and other ethics committee members think about the idea of researchers providing information to participants around the time their participation stops.

We believe research participants who stop taking part early have particular information needs and deserve good quality information as much as any other participants do. We have run a project aiming to help researchers provide information - covering the right topics, in the right way, at the right time.

You can find the guidance we have produced at the following link, as well as a full protocol for this survey (on the 'About this work' page), if you want to see it: https://ctru.leeds.ac.uk/information-to-support-participants-who-stop-taking-part/. Please note that we do not need anyone to read the guidance before completing this survey, but please feel free to look at it if you want to.

This survey has obtained ethical approval from the University of Leeds School of Medicine Research Ethics Committee (reference MREC 22-007). We have made the Health Research

Authority aware of the survey and they have confirmed that it does not need their formal approval before going ahead.

How long will the survey take?

From our testing of this survey, we believe it will take you around 10-15 minutes to complete. However, this will depend on how much detail you want to give in your responses.

You should also know that the survey involves a little bit of reading - this includes within the survey itself where we will ask you to read a couple of paragraphs and give your views in response, and also the answers to the additional questions below (which you should read through if you want to).

Will the survey ask for any identifiable information?

None of the questions ask you to enter any information that could identify you. Please do not enter any identifiable information into the free text questions, either. If you do, please be reassured that we will not keep it.

Although it is very unlikely, there is a small chance that members of the project team at the University of Leeds could identify you in some situations, for example inadvertently based on your survey responses or because you choose to identify yourself to us. If this happens, we will make sure all information that could identify you is kept private (with access only by the Project Lead or other members of the project team at University of Leeds if there is a good reason for them to have access). We will make sure all data collected as part of this survey is protected, including any information that might identify you. See "What will happen to the data that I enter?" below for more information on this.

At the end of the survey, you will have the chance to add your contact details to an optional mailing list to hear about the results of this survey, but this will not be linked to any of the answers you give.

Consenting to take part in this survey

If you decide to complete this survey, we will take this to mean you have consented to take part, based on the information given here. At the bottom of this page, there is a question asking you to confirm you are happy to take part.

More information and other ways to complete the survey
Below, you can find some answers to other questions you may have. Please read through as much or as little of this additional information as you like.

If you have any other questions about this survey, please do not hesitate to get in touch with the lead researcher Will Cragg at w.cragg@leeds.ac.uk. The project supervisor is Rebecca Walwyn. Will and Rebecca both work at the Clinical Trials Research Unit at the University of Leeds.

If you are interested to contribute to this, but might find it difficult to complete an online survey for any reason, please contact Will to find out about other options.

What will the questions be about?

There are 8 main questions in the survey. 3 of these are category questions and 3 are open questions (free text). 2 others ask you to explain your answers to 2 of the category questions (if you want to). We do not intend to report or publish any direct quotations from the open (free text) questions.

The topic of the survey is researcher communication with participants who stop taking part in studies early. The questions will ask your general view on this idea, whether or not you've seen this sort of communication done before, and what you think the implications are for REC review and oversight.

There are also a few questions about you, just so we can understand who has taken part in the survey. The answers to these questions won't identify you, and in any case they are optional, so you can skip them if you want to.

Should I provide a response for myself or on behalf of my REC?

Please answer for yourself, not on behalf of your REC or anyone else. It's fine for several people from the same REC to complete the survey.

Can I skip any of the questions?

Yes, you can skip any questions. All the questions are optional (apart from the first two below about eligibility and consent) and so can be skipped if you don't want to answer them for any reason.

Can I save the survey and come back to finish it later?

Yes, you can save the survey and return to it later, though we would encourage you to complete it in one go if you possibly can.

If you do want to save and return, there is a link at the bottom of each page ("Finish later"). If you click this, you will receive a link by email to return to the survey later. This does not involve the research team having a copy of your email address.

What do you mean by 'stop taking part early'? Do you mean 'withdrawn consent'?

We mean any research participants who stop taking part before their participation was originally due to end. This does include participants who withdraw consent to take part, but also participants who stop taking part because they lose contact with the researcher (sometimes called 'lost to follow-up' by researchers).

It also includes cases where some aspects of participation stops because a doctor or someone else decides it's in the participant's best interests for it to stop (usually in the context of stopping receiving treatment/intervention involved in the research).

We are talking about communication with participants themselves, so things like participants losing capacity, or dying during a study, are not in our scope because researchers in those cases could only be communicating with others (e.g. family or carers).

What will happen to the data that I enter?

We will not ask for any information about you that could identify you, and please do not enter any. If you do enter anything identifiable, we will remove it before doing anything with the survey data.

There is a chance to join a mailing list at the end of the survey if you want to receive the survey results, but this will be entirely separate from the survey, and we will not link your details with the answers you give.

Your survey responses will be stored securely at the Clinical Trials Research Unit at the University of Leeds. For more information about how the University of Leeds protects research data, please go to https://dataprotection.leeds.ac.uk/wp-content/uploads/sites/48/2019/02/Research-Privacy-Notice.pdf.
Until we have finished collecting and analysing survey responses, your answers will be stored in the Jisc Online Surveys platform. This means that for that time, the data will be stored in the Republic of Ireland in the European Union. This means the data will be protected just as well as it would be in the United Kingdom (because data protection in the EU is considered 'adequate' by the UK government). Please see this page for more information on this: https://www.onlinesurveys.ac.uk/help-support/online-surveys-security/

When the survey is finished, we will make the responses available for other researchers to request to use it for further research. We would only share the survey responses for valid additional research projects, and only in such a way that there is no way the researchers could identify anyone from the survey responses.

What will happen to my survey answers and how can I hear about the results of this survey?

We intend to publish the results of this work in a peer-reviewed scientific journal. It will not be possible to identify you or any other survey respondents from this publication.

At the end of this survey, you can choose to join a mailing list to receive a copy of the results, if you would like. We will also share the results with relevant organisations and groups, so you might find out that way as well.

We may also use the results of the survey to update our guidance. We may use the same mailing list to explain what changes we made in response to the feedback we received.

How long will you keep the survey responses and will you share them with anyone else?

We will keep the survey responses for at least 5 years after publication of the results of this project. After this, once the data is no longer useful for research purposes, we will securely destroy it.

We will only share data with people outside of the project team for valid additional research projects, and only in such a way that there is no way they could identify anyone from the survey responses.

Is there any payment or incentive for completing this survey?

There is no money or incentive available for the time you give completing this survey. If this might be a problem for you, then please bear it in mind when you decide whether or not to complete the survey.

Can I share the survey with other REC members?

Yes! Please feel free to share it with anyone else who might be eligible and interested to help.

Checking you are eligible for the survey and happy to take part

This survey is for current members of UK Research Ethics Committees managed by the Health Research Authority's Research Ethics Service. It is for members involved in reviewing and making decisions about applications, but not those who only perform administrative duties. Does this apply to you?

You can still choose 'Yes' if you are having a break from your REC duties but may return. If you have been a REC member in the past but now cannot return (e.g. because you have had the role for the maximum allowed period, or you have formally ended your membership), you are unfortunately not eligible for this survey. A list of the applicable RECs is available here: https://www.hra.nhs.uk/about-us/committees-and-services/res-and-recs/search-research-ethics-committees/

- Yes, I am currently a decision-making member of an HRA-managed REC
- No, I am not currently a decision-making member of an HRA-managed REC

By completing and submitting responses to this survey you are consenting to take part. Please confirm you understand this by answering this question.

- I consent to take part in this survey

[If ineligible]

Unfortunately, you are not eligible for this survey

Unfortunately, you are not eligible for this survey as you are not currently a decision-making member of an HRA-managed REC. Thank you for your interest all the same. You may still join the mailing list to find out the results of this survey when they are ready, if you want to. Go to the next page to find out more.

[If eligible]

Your views on researcher contact with participants who have stopped taking part

Please read carefully through the following explanation as the next question will ask for your views on it.

This survey is being done to get more feedback on a University of Leeds project about providing information to participants who stop taking part in research studies before they were original due to stop (including those who 'withdraw' and those who stop under other circumstances). You can see the main project output here : https://ctru.leeds.ac.uk/information-to-support-participants-who-stop-taking-part/

The rationale for the project is that people who stop taking part in research studies early may have particular information needs. Although they will get some information at the start of the study about what would happen if they stopped taking part early, this may not prepare them fully for when it actually happens. Ending participation can be a stressful experience and participants can sometimes feel unsupported or even 'abandoned'. While there is some existing guidance about providing information to participants at the end of a study, there is very limited guidance about providing information to participants who stop their participation early.

Information specifically useful to this group could include:

- a) clarity about exactly how their participation has changed,
- b) reassurance that they have made an important contribution to the research, despite stopping their participation,
- c) reminders about what stopping early means for them and for any information and biological samples they have given, and
- d) information about what will happen next from their point of view, particularly regarding their care.

How much do you agree with the above rationale?

- Strongly agree
- Somewhat agree
- Not sure / mixture of agreement and disagreement
- Somewhat disagree
- Strongly disagree

Please explain your answer. [Free text]

Have you ever been asked to provide an ethical opinion on a written end of study communication specifically for participants who stop taking part early? (e.g. a 'withdrawal information sheet' or similar)

- Yes
- No
- Not sure / other

Would you have (or have you had) any concerns about approving the use of such written communications? [Free text]

What would you expect to have been considered and addressed by the research team in order for you to approve the use of such written communications? [Free text]

In reviewing any proposed written communications, which of the following do you think would be most appropriate?

- Reviewing the overall proposed communication approach and general information about the sorts of information that would be communicated
- Reviewing the overall proposed communication approach and specific template wording
- Reviewing specific wording proposed to be shared with each individual participant
- Not sure
- Other

If you selected Other, please specify: [Free text]

Please explain your answer. [Free text]

Do you have any other comments on how such communication approaches and materials should be reviewed by ethics committees? Please add details here, if so: [Free text]

About you

All of the questions on this page (and in the survey in general) are optional. But if you are happy to tell us a bit about you, it will help us understand who has taken part in the survey.

Which region is your REC in? (If you could answer about more than one REC, choose your current one or your main one, if possible)

- England – East Midlands
- England – East of England
- England – London
- England – North East
- England – North West
- England – South Central
- England – South West
- England – West Midlands
- England – Yorkshire and the Humber
- Northern Ireland
- Scotland
- Wales
- Not sure / none of the above

How long have you been a REC member (on any REC)?

- Less than 1 year
- 1-5 years
- 6 or more years
- Not sure / other

What is your role on the ethics committee?

- Chair
- Vice chair
- Lay member
- Lay plus member
- Expert member
- Not sure / other

How old are you?

- Younger than 30 years old
- 30-45 years old
- 46-65 years old
- 66+ years old
- Prefer not to say

How would you describe your gender?

- Female
- Male
- Non-binary
- None of the above categories
- Prefer not to say

How would you describe your ethnicity?

- Asian
- Black
- Mixed or multiple ethnicities
- White
- None of the above categories
- Prefer not to say

Other comments

Do you have anything else to add? [Free text]

Final page

Thank you very much for completing this survey.

If you would like to receive a summary of the survey results when they are ready, please consider signing up to the mailing list. The list will only be used for messages about this survey, and if you do subscribe, you can unsubscribe at any time. The answers you have just given in this survey will not be linked to your contact details. Go to **[link]** to sign up.

You can download a copy of the survey's introductory information **[link added]**

As a reminder, our general guidance on this topic is available at https://ctru.leeds.ac.uk/information-to-support-participants-who-stop-taking-part/. If you have any feedback about the guidance, please feel free to share it with the project lead w.cragg@leeds.ac.uk.
