# Supplementary material for: Views of research ethics committee members on end-of-participation communications for trial participants who stop taking part: a cross-sectional survey study
Source: Trials. 2024 Sep 30;25:636. doi: 10.1186/s13063-024-08465-3 (PMC11440916; doi:10.1186/s13063-024-08465-3)
Supplement: Supplementary file 3 — Additional file 3: Supplement 3 Survey protocol (with invitation message) [file 13063_2024_8465_MOESM3_ESM.pdf]

Ethics committee members’ views on informing research participants who stop taking part early

Survey protocol

V2.0 17 October 2022

IRAS ID: 318373

Main researcher/project lead:

Will Cragg

Regulatory and Governance Affairs Manager

Clinical Trials Research Unit, University of Leeds

w.cragg@leeds.ac.uk

Contents

Background ... 3

Aims ... 3

Overview of Design ... 4

Eligibility ... 4

Recruitment ... 4

Survey design ... 5

Analysis and statistical considerations ... 10

Monitoring and oversight ... 11

Approvals ... 11

Ethical considerations ... 11

Data protection and confidentiality ... 12

Archiving ... 12

Publishing and Disseminating Results ... 13

Data sharing ... 13

Transparency ... 13

References ... 14

Additional information ... 15

Background

The Clinical Trials Research Unit at the University of Leeds has carried out a project, funded through the NIHR's "Efficient Studies" funding call, under the title: "Developing and piloting a template communication to improve information and help elicit preferences in people who stop trial participation early".[1] The main aim of this work was to produce a resource for researchers to help them provide good quality information (i.e. information that is useful, necessary, sensitively communicated and delivered in a timely manner) to support participants during the process of ending their participation in clinical trials and other research.

Participants who stop taking part before their participation was originally due to end (including those who actively 'withdraw', and those who stop participating in other ways) may have specific information needs, particularly as stopping participation early can sometimes be a difficult experience.[2,3] The project was initiated against a backdrop of limited existing guidance for researchers about providing information to this participant group, and the likelihood that sensitivities around participants' right to withdraw consent makes some researchers reluctant to provide information.

Following in-depth work with two separate patient groups (over 20 individuals in total), we have now completed the first version of the project's main output, namely a piece of guidance for researchers on how they can provide information to support research participants who stop taking part before the end of a research study. This is now available online [4] and we are in the process of disseminating it to relevant stakeholders in the UK and beyond.

In developing the guidance, we have been conscious that any participant-facing materials that are created in line with the guidance would need to be approved by a Research Ethics Committee (REC) before they were put into practice. We do not yet know what REC members think about the idea of researchers communicating with participants who have stopped taking part in a study, or how likely they are to approve any participant-facing materials. There is also not existing evidence available to answer this question, though evidence from the related PeRSEVERE project (PRincipleS for handling end-of-participation EVEnts in clinical trials Research) suggests that ethics committee members may have relatively conservative views about participants' right to withdraw consent (compared to other people involved in research). [5]

Aims

We would like to carry out a brief survey to achieve the following aims:

1) To find out how much UK REC members support the general idea of researchers communicating with participants who have stopped taking part in a study.
2) To establish if UK REC members tend to have any reservations about this sort of communication and whether or not we need to amend our existing guidance to help deal with such reservations.

We are interested in hearing a wide range of views, and will consciously prioritise number of responses above the depth of information each response provides. The survey will therefore be relatively short and will be advertised as a brief survey.

Overview of Design

We will carry out a short, cross-sectional survey of UK REC members who review healthcare research applications (i.e. members of committees managed by the Health Research Authority [HRA] Research Ethics Service). The survey will primarily be online, but we will offer other completion routes in order not to exclude anyone who would like to take part but cannot use the online survey for any reason.

Eligibility

Individuals will be eligible to take part in the survey if they are currently a member of an HRA-managed REC in the UK (regardless of how currently active they are, i.e. if someone is taking a break from reviewing research applications but is still a listed member then they are still eligible). Individuals who are no longer REC members, regardless of the length of any prior experience, will not be eligible. Members who perform only administrative roles and are not involved in any committee decision-making will also not be eligible.

We will make clear in the survey information that we expect people to contribute as individuals, rather than on behalf of their REC, though we will encourage people to share the survey if other eligible individuals may not have seen it.

None of the members of the project team are REC members, so there is no scope for this conflict to arise (i.e. we are all ineligible for our survey). Individuals who took part in the survey piloting work (see below) will be eligible to take part in the finalised version of the survey.

Recruitment

How the survey will be shared

We will aim to reach REC members via several routes, with prospective HRA approval in place for any routes where this will be required:

- We will share the invitation directly with RECs via the publicly available email address for each REC. [6] We will ask the administrator (or whoever is otherwise managing the email address) to share with the members of the REC, if they are happy to do so. Alternatively, we may rely on HRA help to disseminate the survey invitation directly to REC members if HRA advises us that this is preferable.
- We will request for a message about the survey to be added to one of the regular ‘bulletins’ that UK REC members receive.
- We will request to share the invitation via relevant UK-based networks in case these reach REC members, e.g. the UK Trial Managers’ Network and the UK CRC Registered CTU Network.
- We will share an invitation message via Twitter, and encourage others to retweet the message on our behalf (including HRA, if they are supportive).
- In general, we will encourage onward sharing of the survey if individuals know of other eligible people.

The email and social media invitation messages that we plan to use are shown in “Additional Information”, below. Please note that the limitations and requirements on a message that could be

included in the REC email bulletins are not known; if the invitation text given below is not suitable for that format, it would be amended only to meet those requirements (e.g. reduced word count).

Reminders

If we share the surveys using the individual REC email addresses (as above), we will track responses to the invitation from individual RECs. For any who confirm they will or will not share the survey, we will not send any further reminders. For any who have not responded after several weeks, we will get in touch again once to prompt them for a response.

We will not be able to send reminders via Twitter, but we may re-share the link several times during the period the survey is open.

In general, we will use accumulating responses to guide further dissemination efforts (see **Monitoring and Oversight**, below).

Survey design

Survey design process

The survey questions have been designed to meet the objectives set out above. The project lead drafted the questions, then refined them in consultation with the rest of the project group (made up of majority patient members).

We have tested a draft version of the survey within the wider project team, and also with the help of three current REC members.

Consent

In line with HRA guidance on proportionate consent, consent to participate in the survey will be presumed to have been given when people choose to complete it. This approach will be made clear in the survey introductory text. Potential respondents will nonetheless be provided with adequate information up-front in order to make an informed decision, including about the purpose of the survey, and including confirmation that participation is voluntary, and that they have a right to stop completing the survey after having started without any negative consequences. Respondents will be able to access a copy of the information to keep, if they want it.

The survey will not ask anyone to provide identifiable personal or confidential data. We will set up a mailing list for individuals to stay in touch with the project (see **Publishing and Disseminating Results**, below), but this will be completely separate to the survey and personal details will not be linked to survey responses.

As the survey will not be collecting any identifiable details about the respondents, once they have submitted their response it will not be possible to delete it because it will not reasonably be possible to identify them from their response. This will be made clear to respondents before they agree to complete the survey. Respondents are free to stop taking part in the survey at any point after having started, and all the survey questions will be optional (apart from the questions about eligibility and consent).

Survey period

The survey period will primarily be driven by the number of responses, i.e. we may leave the survey open for longer in order to allow more responses to be gathered. We will close the survey when we have met the target respondent number, or when very few respondents are continuing to complete it and we are not planning any further promotion.

Survey platform

We will use Jisc's 'Online Surveys' platform [7] to conduct the survey. There are no technical controls to prevent individuals from completing the survey more than once (even from the same computer), but we do not suspect anyone would have strong motivation to do this. Nonetheless, we will perform a check for possible duplicates during analysis.

In order to take an inclusive approach towards those for whom the online option is not suitable, we will offer other routes to complete the survey, specifically:

- Completing a paper copy of the survey and returning it to the project lead for data entry (this would include using the paper copy to confirm consent to participate);
- Completing the survey over the phone (or via a Microsoft Teams meeting) while the project lead performs data entry (this would include reading through all the pre-survey information before they agree to take part);
- Any other feasible method if none of the previously listed options are suitable.

However, we expect the Online Surveys platform to suit most potential respondents due to its in-built accessibility features [8], which the service provider has confirmed also apply to the survey as well as the website in general. If any individuals do complete the survey via any of these alternative methods, there is a chance that this will affect the answers they give. We will therefore summarise the number of respondents using the alternative methods when we report the results of the survey.

For anyone who chooses to complete the survey via one of the alternative methods mentioned above, the Project Lead will inevitably need to obtain their contact details (see **Data protection and confidentiality,** below). These details will be provided voluntarily, and previous advice from the Information Commissioner's Office indicates a proportionate approach can be taken to meet the data protection transparency principle in these sorts of cases. Respondents will nonetheless be informed and reassured that their details will not be retained for longer than needed and will not be linked to or stored with their survey responses.

Survey content and questions

The survey content will comprise the following:

- Introductory content:
    - The brief introduction will reiterate the aims of the survey, explain what is involved in completing it and how long it is likely to take, and what will happen to the results. It will also make clear that participation is completely voluntary, with no negative consequences of not taking part, or of starting to complete the survey then stopping before the end.

- It will give advance notice of the topics covered in the survey so people know what to expect (as a way to encourage completion of all questions). It will confirm that all questions will be optional and can be skipped.
- It will confirm that we do not expect anyone to enter any identifiable information (except for their details into the optional mailing list, which will be kept completely separate to their responses), but nonetheless provide information about how the data they provide will be used, via the University of Leeds research transparency policy. [9]
- It will confirm that individuals should reply for themselves, not on behalf of anyone else.
- It will explain that completion of any of the survey will be taken as implied consent to complete it.
- It will confirm that, as responses cannot be linked back to individuals, there is no way to remove responses once they have been entered.
- It will confirm that there will be no financial incentive or other payment available for completing the survey.
- It will confirm that relevant approvals are in place for the survey.
- Contact details of the lead researcher will be given in case of any questions or issues.

- Initial questions to check eligibility and confirm people are happy to continue and take part (based on the survey introduction).

- We will explain the rationale for the wider project and ask respondents how much they agree with it.
    - "This survey is being done to get more feedback on a University of Leeds project about providing information to participants who stop taking part in research studies before they were original due to stop (including those who 'withdraw' and those who stop under other circumstances). You can see the main project output here: https://ctru.leeds.ac.uk/information-to-support-participants-who-stop-taking-part/"
    - "The rationale for the project is that people who stop taking part in research studies early may have particular information needs. Although they will get some information at the start of the study about what would happen if they stopped taking part early, this may not prepare them fully for when it actually happens. Ending participation can be a stressful experience and participants can sometimes feel unsupported or even 'abandoned'. While there is some existing guidance about providing information to participants at the end of a study, there is very limited guidance about providing information to participants who stop their participation early."
    - "Information specifically useful to this group could include a) clarity about exactly how their participation has changed, b) reassurance that they have made an important contribution to the research, despite stopping their participation, c) reminders about what stopping early means for them and for any information and biological samples they have given, and d) information about what will happen next from their point of view, particularly regarding their care."
    - How much do you agree with the above rationale?
        - Strongly agree
        - Somewhat agree
        - Not sure / mixture of agreement and disagreement
        - Somewhat disagree
        - Strongly disagree

- Please explain your answer [free text]

- We will ask respondents about their experiences and views on ethical approval of researcher communications with participants who stop taking part:
  - Have you ever been asked to provide an ethical opinion on a written end of study communication specifically for participants who stop taking part early? (e.g. a 'withdrawal information sheet' or similar)
    - Yes
    - No
    - Not sure / other

  - Would you have (or have you had) any concerns about approving the use of such written communications? [free text]

  - What would you expect to have been considered and addressed by the research team in order for you to approve the use of such written communications? [free text]

  - In reviewing any proposed written communications before they are used, which of the following do you think would be most appropriate?
    - Reviewing the overall proposed communication approach and general information about the sorts of information that would be communicated
    - Reviewing the overall proposed communication approach and specific template wording
    - Reviewing specific wording proposed to be shared with each individual participant
    - Not sure
    - Other (specify)
  - Please explain your answer.
  - Do you have any other comments on how such communication approaches and materials should be reviewed by ethics committees? Please add details here, if so:

- We will ask about the characteristics of the respondents (nonetheless via categorised, non-identifiable responses). These will help us understand who has completed the survey and how diverse they are in terms of their characteristics, and will let us explore whether different sorts of people have provided different answers. All the questions will be optional, as they will for the whole survey (except for the eligibility and consent questions). The planned questions are as follows:

  - Which region is your REC in?
    - England – East Midlands
    - England – East of England
    - England – London
    - England – North East
    - England – North West
    - England – South Central
    - England – South West
    - England – West Midlands
    - England – Yorkshire and the Humber

- Northern Ireland
- Scotland
- Wales
- Not sure / none of the above

- How long have you been a REC member?
    - Less than 1 year
    - 1-5 years
    - 6 or more years [1]
    - Not sure / other

- What is your role on the ethics committee?
    - Chair
    - Vice Chair
    - Lay member
    - Lay plus member
    - Expert member
    - Not sure / other

- How old are you? (Optional)
    - Younger than 30 years old
    - 30-45 years old
    - 46-65 years old
    - 66+ years old
    - Prefer not to say

- How would you describe your gender? (Optional)
    - Female
    - Male
    - Non-binary
    - Neither of the above categories
    - Prefer not to say

- How would you describe your ethnicity?
    - Asian
    - Black
    - Mixed or multiple ethnicities
    - White
    - None of the above categories

[1] HRA guidance suggests that individuals can only be a REC member for a maximum of 10 years (https://www.hra.nhs.uk/about-us/committees-and-services/res-and-recs/become-rec-member/information-potential-research-ethics-committee-members/). We might on this basis make the category here 6-10 years, but as we do not know how strictly this limit is enforced, there may be individuals who might want to say e.g. 11 years (or they may have been a member at two different times in their lives for longer than this). In order to avoid this added complication, we have chosen 'more than 6 years' as a way to differentiate those with the greatest level of experience.

    - Prefer not to say

- Any final comments ("Do you have anything else to add?")

- A thank you message and invitation to join the associated mailing list (with up-front confirmation that personal details will not be linked to survey responses; see **Publishing and Disseminating Results, below** for more on the mailing list). On the final page, we will also provide another link to our guidance and invite any more specific feedback on it. The final page will also give respondents another chance to download the survey's introductory text if they want to.

Analysis and statistical considerations

Sample size

We will not perform a formal sample size calculation as we are not performing any comparative statistical analyses. Instead we can set out an arbitrary target that we believe will give us a strong conclusion.

Previous contact with the HRA indicates that there are around 800-900 members of HRA-managed RECs. We therefore consider that a response rate of around 25% (i.e. ≥200 respondents) would be good, and 50% (i.e. ≥400 respondents) would be excellent. We will also aim for a good balance between expert and lay members and from different UK regions. If necessary, we will adjust the recruitment approach while the survey is open to aim for more respondents from particular groups.

Although not required to perform a sample size calculation, for the purposes of interpreting the results of the work, the following question will nominally be the primary outcome measure: "How much do you agree with the above rationale?" This is because a) it is quantifiable, and b) it addresses the fundamental point of our guidance, i.e. the need to inform participants. If our survey discovers that REC members have concerns about the specific approach taken, this will be useful to know, but if they nonetheless support the more general motivation for the guidance (for example) then this will mean the overall aim is the right one.

Data management

During analysis, we will highlight any inconsistent or illogical results (e.g. free text comments that seem to conflict with responses to quantitative questions) and discuss and handle these appropriately. As mentioned above, we will also check for any evidence that respondents have completed the survey more than once (e.g. if there are any very similar entries).

We will include missing data in our descriptive statistics. We will not impute any data, except potentially in the situation where a qualitative response has been left missing but the related free text response unambiguously indicates an answer (e.g. "I completely agree with this"). Any such imputation will be clearly documented and reported in the final project outputs.

All survey responses will be downloaded prior to analysis and stored in a secure file location within the Clinical Trials Research Unit, University of Leeds. The project lead will scan the free-text responses for any identifiable information added (despite the instructions not to do this) and remove it immediately if any is found.

Analysis

The analysis population will be all respondents who confirm they are eligible and happy to take part, and who add and save any responses into the survey.

All quantitative questions will be summarised in terms of proportions for each category answer, including missing responses. We will use 95% confidence intervals for the population proportion to indicate the relative size of differences in proportions. The primary outcome question about overall agreement with our project's rationale will also be summarised using the median, after having converted the responses to numbers 1-5 (with 'strongly disagree' being 1, and 'strongly agree' being 5).

Responses to the free text fields will be summarised via inductive analysis, working without a pre-existing framework to categorise each comment initially at a granular level (i.e. based on its specific contents) then combining these categories into broader themes. We have chosen this approach as we have neither a prior framework to work with, nor any strong rationale to make prior assumptions about the sorts of comments we will receive. Coding will be double-checked by another member of the overall project group for a random 10% sample of respondents' free text responses. Any corrections will be made as required following this checking, and further checking will be carried out if, in the view of the checker, there are serious concerns about the accuracy or consistency of coding.

We will carry out exploratory analysis of the quantitative and qualitative responses to look for differences in answers given by different subgroups, and use the population proportion confidence intervals to help understand the strength of any differences.

Monitoring and oversight

While the survey is open, the project team will regularly review overall response numbers, as well as the number of respondents who identify themselves as 'expert' and 'lay members' (including both 'lay' and 'lay plus' members). We will use these accumulating response figures to guide further dissemination efforts.

The survey has been designed by members of the overall project group (see **Background**, above), who will also help with more general survey oversight and with interpretation of the survey results. Patient contributors make up the majority of the project group.

Approvals

We will obtain ethical approval from the School of Medicine Research Ethics Committee and HRA approval before accepting any responses to the survey.

Ethical considerations

We do not anticipate that this survey will raise any significant ethical issues. Respondents will be asked general questions about their views on a broad topic in clinical research and about ethical review

processes. Although we cannot exclude some respondents being upset if particular questions remind them of something in their past, no sensitive information is collected in these questions. Some of the demographics questions could be considered sensitive, but we have consciously aimed to word them generally and non-intrusively. Survey completion is completely voluntary and optional, with no negative consequences of skipping questions or abandoning the survey partway through.

Data protection and confidentiality

No identifiable personal or confidential data will be requested in the survey (the mailing list mentioned in **Publishing and Disseminating Results** below will be entirely separate), and the introductory text will remind people not to enter anything identifiable in any free text fields. Should something identifiable be added by a respondent, it will be deleted by the project lead before the data is shared with anyone else.

However, we cannot rule out some of the data collected in the survey constituting personal data *to the project lead only* in at least the following scenarios:

- An individual contacts the project lead to say that they are going to, or just have, completed the survey, and to talk about certain answers they gave;
- The project lead inadvertently makes links between the survey and the mailing list (despite not planning to link the two together), e.g. based on the date of completion;
- For any individuals who do want to provide responses via a different route than the survey, it will not be possible for them to complete the survey anonymously (see **Survey platform**, above).

In all of these hypothetical cases, only the project lead will be able to identify the survey respondents from the survey data, so the data would only be personal data in his possession; no one else within or outside the project team will be able to identify individuals from the data at any stage.

For any personal data that is collected (including the above scenarios from the project lead's point of view, and for the mailing list), the University of Leeds will be the data controller. Respondents will in any case be informed of the fact that their responses to the survey (but not the contact details they add to the mailing list) will be stored in the Republic of Ireland, due to Jisc's data storage arrangements.[10]

Archiving

Survey responses will be collected within Online Surveys at University of Leeds (including for the other response routes mentioned in **Survey design**, above). Responses will be downloaded and stored in a secure location at the Clinical Trials Research Unit (CTRU), University of Leeds. Data shared with other members of the project group for review will not contain any information that could identify any survey respondents, and will be sent appropriately securely.

In line with University of Leeds data retention schedule,[11] survey data will be retained for at least 5 years after publication of the main planned peer-reviewed paper about this work.

Publishing and Disseminating Results

Publication

We intend to publish the results of the survey as part of a broader publication about this project. We have consulted the CROSS checklist [12] in developing this survey protocol and we will adhere the CROSS requirements in reporting the results as well. The published results will not identify or single-out any individual responses.

We will ensure we comply with HRA's timelines for making research results public if HRA informs us that this timeline (i.e. 1 year after the end of the study) also applies to this work.

Sharing results with respondents

We will set up a simple, optional mailing list for the purposes of sharing the results of the survey with the respondents. This may include summarising any themes in feedback on our project more generally, and what we have done in response to this feedback.

The mailing list will be managed by the project lead. Email addresses will be collected using Microsoft Forms, and during that time the information will be stored in the University of Leeds' Microsoft cloud platform. Once the survey is closed, the data will be downloaded to a secure location within the Clinical Trials Research Unit, University of Leeds, and the information in Microsoft Forms will be deleted. Individuals will receive information about how their data will be handled before they enter any details into the Microsoft Form. The mailing list and its data will be retained only for as long as it is in use to send messages about the project. After this time, it will be securely destroyed. Individuals will be able to unsubscribe at any time by contacting the project lead, and all messages sent to the mailing list will remind individuals of this possibility.

We will also make it possible for individuals to give a different contact method if they do not want to receive the results via email, for any reason. In this case, their contact details will be collected directly by the project lead and stored securely within the Clinical Trials Research Unit.

We will also request that the HRA shares a link to the results via a more general route, e.g. the bulletin newsletters for members, as another way for interested individuals to hear the results.

Data sharing

Data collected as part of this work will be available for further research on reasonable request, once any planned peer-reviewed publications are released. Data will be shared according to a controlled access approach, in line with CTRU policies. Data will only be shared in such a way that means there is no chance any individuals could be identified.

Transparency

This project is not eligible for registration on clinical trials registries such as ISRCTN or clinicaltrials.gov. However, for transparency, we will make a copy of this consultation plan available on the website where the project guidance has been published [4] before opening the survey to responses.

References

1. https://www.nihr.ac.uk/documents/ad-hoc-funding-calls-for-ctu-projects/20141#2020
2. Ulrich, C. M., Knafl, K., Foxwell, A. M., Zhou, Q., Paidipati, C., Tiller, D., Ratcliffe, S. J., Wallen, G. R., Richmond, T. S., Naylor, M., Gordon, T. F., Grady, C., & Miller, V. (2021). Experiences of Patients After Withdrawal From Cancer Clinical Trials. JAMA Network Open, 4(8), e2120052–e2120052. https://doi.org/10.1001/JAMANETWORKOPEN.2021.20052
3. Cox K, Wilson E, Arthur A, Elkan R, & Armstrong S (2005). A randomised controlled trial of nurse-managed trial conclusion following early phase cancer trial participation. British Journal of Cancer, 93(1), 41–45. https://doi.org/10.1038/SJ.BJC.6602675
4. https://ctru.leeds.ac.uk/information-to-support-participants-who-stop-taking-part/
5. https://ukcrc-ctu.org.uk/page-persevere/
6. https://www.hra.nhs.uk/about-us/committees-and-services/res-and-recs/search-research-ethics-committees/
7. https://www.onlinesurveys.ac.uk/
8. https://www.onlinesurveys.ac.uk/accessibility/
9. https://dataprotection.leeds.ac.uk/research-participant-privacy-notice/
10. https://www.onlinesurveys.ac.uk/help-support/online-surveys-security/
11. https://dataprotection.leeds.ac.uk/data-protection-and-personal-data/#how-long-can-I-keep-personal-data
12. Sharma et al. A Consensus-Based Checklist for Reporting of Survey Studies (CROSS). *Journal of General Internal Medicine* **36,** 3179-3187 (2021). https://link.springer.com/article/10.1007/s11606-021-06737-1

Additional information

Email invitation message

Short survey: should researchers be able to communicate with research participants who stop taking part in studies early?

We (a group of patients and University of Leeds researchers) invite all UK REC members to take part in a short survey about researcher communication with research participants. Our testing indicates that it might take 10-15 minutes to complete it, although this does depend on how detailed your responses are.

The survey relates to a wider project about how best to support research participants who stop taking part early (including those who withdraw consent and who stop in other circumstances).

We have developed some guidance for researchers about how to ensure these research participants get access to good quality information to support them when they are ending their participation early.

Any such researcher contact would clearly need approval and oversight by an independent research ethics committee. We therefore would like to know what you, as a REC member, think about the idea of researchers providing information to participants around the time of their stopping participation. This includes what level of review you would suggest for communications, any issues you would want to be addressed and how you would expect communication materials to have been developed.

The survey only has 8 main questions (3 category questions, 3 open questions and 2 other questions asking you to explain your answers to the category questions). All questions are optional.

We hope you will consider taking part, as all responses will be gratefully received. You can find the survey, and more information about what taking part will involve, at **[add final link].** The initial closing date will be **[date]** but we may extend this if responses are still coming in.

Please feel free to pass to other REC members who might like to take part. Please note that we are requesting responses from individuals, not for each REC, and more than one response per REC is allowed. Only current REC members are eligible (including those on a break who could in theory return).

If you have any questions, or if you would like to take part in this but cannot use the Online Surveys format for any reason, please contact the project lead Will Cragg at w.cragg@leeds.ac.uk.

[Add details of confirmed approvals once in place]

Twitter message template

Are you a current HRA REC member? We'd like your views abt researchers providing info to research participants when they stop taking part early. Survey's anonymous & likely to take 15 mins or less. Full invitation in image. Link is here & please do share: **[link]**
